# The effect of the introduction of livestock on the erosion of alpine soils: a comparison of five dating techniques applied to sediments of the Australian alpine Blue Lake

**DOI:** 10.1007/s10933-023-00284-x

**Published:** 2023-05-08

**Authors:** Patrick De Deckker, Gary J. Hancock, Jon M. Olley, Shawn Stanley, Geoffrey Hope

**Affiliations:** 1 Research School of Earth Sciences, The Australian National University, Canberra, ACT 2601 Australia; 2 Formerly of CSIRO Land and Water, PO Box 1666, Canberra, ACT 2601 Australia; 3 School of Environment and Science - Forensics and Archaeology, Griffith University, Nathan, Qld 4111 Australia; 4 Formerly of Department of Geology, The Australian National University, Canberra, ACT 2601 Australia; 5 Division for Ocean Affairs and the Law of the Sea, Office of Legal Affairs, United Nations, New York, NY 10017 USA; 6 Canberra, Australia

**Keywords:** AMS ^14^C, Optical dating, ^210^Pb and ^137^Cs geochronology, *Pinus* pollen, Erosion, Land management

## Abstract

^210^Pb and ^137^Cs dating of bulk sediments obtained from the alpine Blue Lake, located in the Snowy Mountains of southeastern Australia, was applied here to date recent lacustrine sediments. In addition, the presence of *Pinus* pollen (a taxon introduced in Australia about 150 years ago) down to a sediment depth of 56 cm in the core is used to obtain a chronology for the upper part of the core. Accelerated Mass Spectrometry radiocarbon dates obtained from organic muds from the same core do not agree with the chronology constructed using the three other dating techniques. In addition, optically stimulated luminescence (OSL) dating of single quartz grains, from sediment-core samples collected from the same lake, was applied to date recent lacustrine sediments. The optical age of 185 ± 20 years for a sample at 60–62 cm depth, and 470 ± 50 years at 116–118 cm depth are well over 1000 years younger than the ages inferred from radiocarbon dates. We therefore infer that the ‘old’ radiocarbon ages result from carbon stored for considerable time within the catchment prior to its transport and deposition on the lake floor. As plant decomposition occurs at much slower rates in high altitude environments, these results bring into question the veracity of previously published radiocarbon dates from Blue Lake and alpine lake sediments in general. The deposition ages inferred from the ^210^Pb-^137^Cs and OSL dating, and the first appearance of *Pinus* pollen, indicate that for the 100-year period after European settlement (from the mid 1800s to early 1900s) the sediment-accumulation rate increased by a factor of about 2, from 0.19 ± 0.01 cm yr^−1^ to 0.35 ± 0.02 cm yr^−1^. In the 1900s the accumulation rate increased further to 0.60 cm yr^−1^. The accumulation rate was particularly rapid in the 20-year period from 1940–1960, reaching a rate 18 times higher than the pre-European rate in the mid-1950s. The increase in sedimentation rate is attributed to changes in land use resulting from European activities in the lake catchment, primarily through sheep and cattle grazing in the Blue Lake catchment.

## Introduction

The timing of environmental change in Australia and elsewhere, particularly during the Holocene period, has been inferred using radiocarbon dates of material recovered from lacustrine cores (De Deckker 2022). Until the early 1980s, conventional radiocarbon-dating techniques were used. However, because a substantial amount of material was needed for the gas counting or liquid scintillation methods, accurate dating of specific, short-term events could rarely be obtained. The advent of accelerated mass spectrometry (AMS) (Muller 1977) permitted the analysis of much smaller samples which helped improve the resolution of the dating of environmental change. Narrower horizons in lacustrine cores could be analysed and dated. Despite this improvement, many irreconcilable data based on radiocarbon dates remain, especially where different carbon fractions from the same sample have been dated (Bowler et al. (1986) and Gillespie et al. (1991)).

It has been recognised that organic material may have formed much earlier than the time of its final transport and deposition. Bajard et al. (2017) documented the effects of agro-pastoralism activities that go back as far as 4300 calibrated years before present (cal yrs BP) on Lake Verney, located at 2188 m above sea level (a.s.l.) on the Italian side of the Petit Saint-Bernard Pass in the French-Italian Alps. They showed that soil erosion significantly increased during the Roman Period due to sheep grazing, and further increased during the Middle Ages due to cattle grazing. In another study in the northern French Alps, Bajard et al. (2020) identified that soil erosion is not only linked to precipitation levels, but also changes in land use. They clearly identified that human activities, such as deforestation and pastoralism, also during the late Roman Period and later in the Middle Age, principally with cattle grazing, further engendered soil erosion. It is therefore important to test chronologies based on radiocarbon dates, not just by multiple dating of subsamples and by comparison between dates on different organic and inorganic fractions, but also by comparison with other dating techniques.

The high country in Australia is defined by what is called the Australian Alps in the Snowy Mountains, and encompasses Australia’s highest peak Mount Kosciuszko (2228 m a.s.l.). The Alps, including the Australian mainland’s snow country, covers some 25,000 km^2^ above 1370 m, and represents only 0.5% of the continent. As early as 1906, the Kosciuszko National Park came into existence as the National Chase Snowy. In April 1944, the Kosciusko State Park was eventually declared and in 1967, after much community and governmental debate, the Kosciuszko National Park was formally declared. Its surface area is 6980 km^2^. The incentives for claiming such a status were the combination of habitats and plant communities that were among the richest in Australia. There are over 120 taxa of herbs which make the most extensive and diverse herbfield in the alpine area (Costin et al. 1979; Scherrer and Pickering 2005). Records show that graziers were taking sheep and cattle into and across the mountains in the early 1820s in search of new pastures which were free of drought and disease. As a result of major concerns by various governments and individuals dealing with soil erosion and vegetation damage by the 1950s, cattle were excluded from the highest summits including Mounts Bogong, Hotham, Loch and Feathertop to protect the water catchment and alpine vegetation (Good 1989).

Although the time scales are very different compared to those Italian and French lakes, here we aim to determine the effects of the introduction of livestock in the Australian Alps ~ 200 years ago on sedimentation rates in Blue Lake, Australia’s highest lake, as well as determine changes that may have operated since livestock was removed from the area. In this study, we have used optical dating of single grains of quartz, ^210^Pb and ^137^Cs dating of bulk sediments, AMS radiocarbon dating of organic lake muds (here called gyttja), and the first appearance of *Pinus* pollen (an exotic brought to Australia by Europeans) to date sediment samples from the alpine Blue Lake in the Snowy Mountains of southeastern Australia.

## Materials and methods

### Study site

**Fig. 1:**
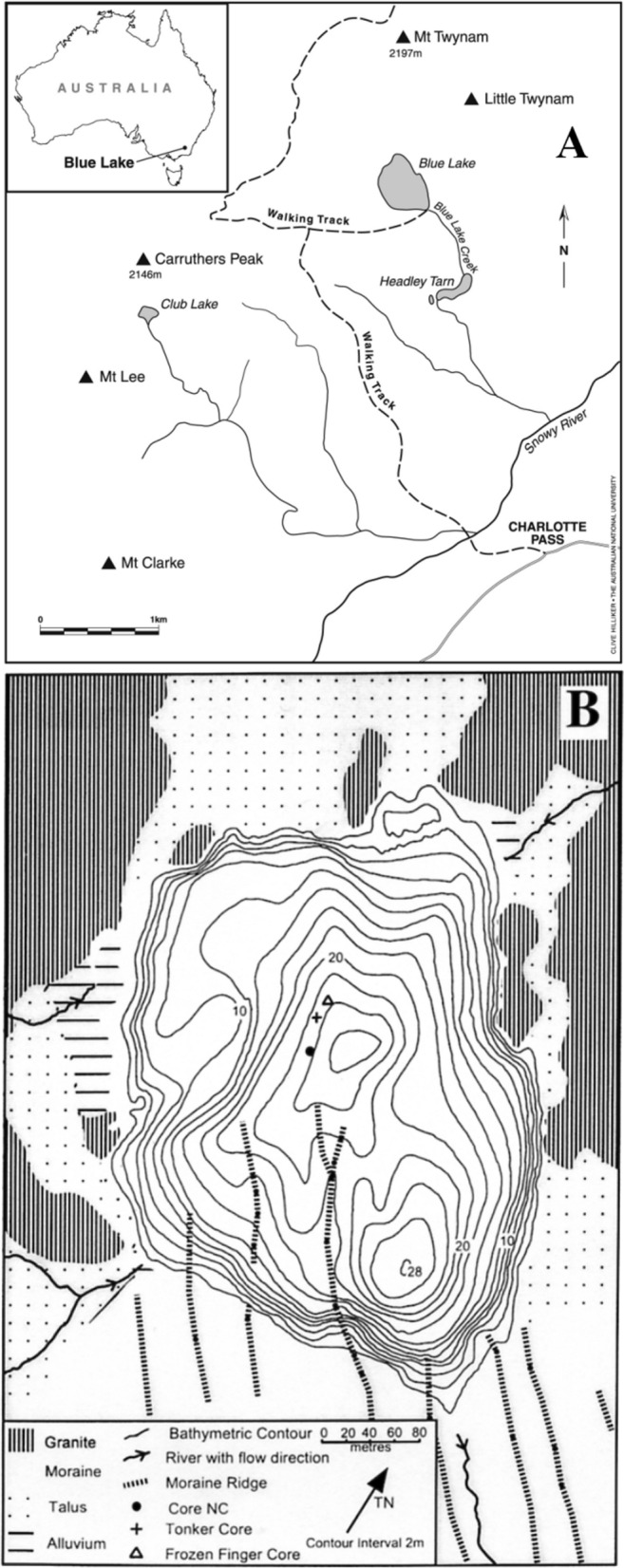
Location map of Blue Lake in the Snowy Mountains, New South Wales. **A** shows a regional map with the location of Blue Lake and other sites in the area. **B** shows a detailed map of Blue Lake with the location of the various cores, the regional geology and bathymetric contours in 2 m intervals

Blue Lake is one of only a dozen deep, freshwater lakes in Australia. It is located at 148° 19' E, 36° 25'S near the highest mainland mountain, Mount Kosciuszko (Fig. 1a). The 28-m deep lake is at an altitude of 1930 m and is above the tree line, which occurs in this area at about 1780 m a.s.l. It is frozen in winter, and is therefore dimictic (Raine 1982; Burgess et al. 1988). It is almost trapezoidal in shape (540 m long and 360 m wide), and has only a small catchment area (1.9 km^2^; Fig. 1b). Blue Lake is the only Australian mainland lake which formed as a result of glacial erosion followed by glacial melting (recognised by David et al. (1901), and subsequently investigated by Dulhunty (1945) and Galloway (1963)). The area was glaciated at the height of the Last Glacial Maximum (Barrows et al. 2001), which was also a period of extensive aridity for most of Australia (De Deckker et al. 2020). There was only sparse feldmark vegetation in the lake catchment at the time of deglaciation about 14,600 cal yrs BP (Raine 1974).

The lake’s catchment consists of two principal rock types, Carboniferous granites and Ordovician metasediments (Fig. 1). The regolith is generally thin and consists of the weathered byproduct of the granites and metamorphosed shales, as well as black, organic-rich soils. Numerous rock outcroppings occur throughout the catchment. The vegetation belongs to the “Tall Alpine Herb Field” plant community (Costin et al. 1979), and consists principally of heaths, alpine daisies, grasses and the sparsely distributed, dwarf and slow growing *Podocarpus lawrencei* (= mountain plum-pine) with an average growth of 0.25 mm yr^−1^ (Costin et al. 1979). Precipitation in the area is estimated to be 1770 mm yr^−1^, and the mean annual temperature is 4.2 °C, with a range between summer maxima of 16.9 and winter minima of − 5.2 °C. Additional information on the local geomorphology and flora is available in Costin et al. (1979), on the limnology in Burgess et al. (1988), the water chemistry in Williams et al. (1970), on benthos analysis in Timms (1979) and on actuopalynology and Holocene palynology in Raine (1982, 1974). Stanley and De Deckker (2002) discussed a record of regional aeolian activity obtained from a core at Blue Lake.

Sediment cores have been collected from the centre of Blue Lake on three previous occasions: A. Costin obtained a 350 cm core in 1967 using a gravity tube and dated the gyttia at 321 cm to about 6000 radiocarbon yrs BP. In 1972, I. Raine took three Mackereth cores which overlapped to provide a sequence down to 680 cm. Raine’s (1974) record covers the early history of the lake with a basal 250 cm of glacigenic rockflour overlain by 4 m of black algal gyttia. Another Mackereth core NC, 436 cm in length was obtained by C. Barton in 1974 for his study of changes in magnetic field during the Holocene. In both the Raine and Barton cores, the process of anchoring the drum (located at the base of the corer) to the lake floor would have been quite destructive, and consequently the sediment near the sediment-water interface is unlikely to have been recovered. Quite old radiocarbon dates were obtained from near the top of the Costin and Raine cores: 825 ± 100 yrs BP (NZ 586) for 15 cm from the top in the Costin core and 1880 ± 70 yrs BP (ANU 892) at 20–40 cm in the Raine core. The Raine core also showed an age inversion with the sample at a core depth of 79–97 cm giving an age of 900 years younger [920 ± 60 (ANU 1093)] than the sample at 20–40 cm. These dates are uncalibrated.

### Cores

In our study, we used two coring techniques to obtain a complete sediment sample from the sediment-water interface to a sediment depth of 120 cm. Both the frozen finger corer and the tonker core were taken in 1998.

1. The frozen finger technique, which freezes sediments to a tube packed with dry ice, was used to recover material from the sediment-water interface down to a sediment depth of 60 cm. For further details on the technique, refer to Berglund (1986). The frozen core, soon after being taken, was packed on dry ice in a polystyrene box and then taken to the micropalaeontology laboratory in the Department of Geology at the Australian National University where it was sectioned in 1 cm intervals. The sediment–water interface was clearly visible as frozen water was also collected just above the sediment. These sections were stored in plastic bags in a conventional freezer. Subsamples of these individual sections were used for ^210^Pb and ^226^Ra analysis, pollen analysis, extraction of quartz particles for microscopic examination and distinguishing local quartz grains from aeolian-transported ones (Stanley and De Deckker 2002), and for one AMS date on organic gyttia at a sediment depth of 35–36 cm.
2. The tonker coring technique (Neale and Walker 1996) was used to obtain sediment from the surface down to a sediment depth of 120 cm. This technique uses a PVC tube to recover lacustrine sediments that is protected from light. Two sub-samples were taken for dating using optically stimulated luminescence from depths of 60–62 cm and 116–118 cm. Sub-samples were also taken for radionuclide analysis to allow stratigraphic correlation with the frozen finger core, and to provide dose rate estimates for the optical dating (sampling depths are given in Table 1).

**Table 1:** Frozen finger core activity concentrations (Bq kg^−1^) of lithogenic and fallout radionuclides used for ^210^Pb-^137^Cs chronology and stratigraphic correlation with the tonker core

Depth (cm)	Porosity	LOI %	^238^U	^210^Pb	^226^Ra	^210^Pb_ex_	^137^Cs
0–1	0.978			730 ± 26		663 ± 26	
1–2	0.910	0.166	70.4 ± 6.0	361 ± 11	67.1 ± 1.5	294 ± 11	34.7 ± 1.2
2–3	0.901	“	“	251 ± 11	“	184 ± 11	“
3–5	“			268 ± 4	“	201 ± 4	“
5–7	0.901	0.161	71.4 ± 5.8	240 ± 5	67.0 ± 2.0	173 ± 5	11.6 ± 1.1
7–9	0.902	0.249		298 ± 10	59.9 ± 2.0	239 ± 10	
12–14	“	0.249	97.3 ± 5.9	208 ± 7	59.0 ± 1.6	149 ± 8	5.6 ± 0.8
14–16	0.901	0.249		178 ± 5	“	119 ± 6	
18–20	“	0.280	125 ± 8	138 ± 4	53.2 ± 1.9	85 ± 4	11.0 ± 1.0
20–24	“	0.281	125 ± 7	162 ± 13	53.1 ± 2.1	108 ± 13	9.9 ± 1.1
24–26	0.885	0.280	151 ± 18	142 ± 13	58.8 ± 3.3	84 ± 13	11.0 ± 1.3
26–27	“	0.253	134 ± 9	96.8 ± 4.2	52.5 ± 2.8	44.3 ± 5.0	8.4 ± 1.7
28–30	0.883	0.279	134 ± 7	89.0 ± 2.9	54.0 ± 1.0	35.0 ± 3.1	3.8 ± 0.6
32–34	“	0.279	“	79.0 ± 10.0	52.6 ± 1.4	26.4 ± 10.1	
34–35	0.880	0.254	150 ± 13	63.6 ± 2.1	52.5 ± 2.0	11.1 ± 2.9	2.9 ± 0.8
36–37	“	0.264	140 ± 5	63.2 ± 2.1	52.2 ± 2.3	11.0 ± 3.1	2.2 ± 1.0
38–40		0.279	112 ± 6	63.3 ± 2.2	48.2 ± 2.0	15.1 ± 3.0	0.1 ± 1.4
44–45	0.884			69.2 ± 2.7	50.0 ± 1.8	19.2 ± 3.2	
46–48				68.7 ± 2.3	54.2 ± 1.8	14.5 ± 2.9	
49–52				70.0 ± 2.4	49.7 ± 2.1	20.3 ± 3.2	
52–60	0.878			57.8 ± 2.0	49.0 ± 2.0	8.8 ± 2.8	

The symbol “ refers to the value directly above in each column and results from a single measurement spanning more than one depth increment. Blank spaces indicate that no measurement was made at that depth. Uncertainties correspond to 1 standard deviation

An additional core obtained with the use of a Mackereth corer was taken in March 1977 and will also be referred to in the text.

### Dating methods

#### ^210^Pb and ^137^Cs geochronology

^210^Pb and ^137^Cs geochronology was described in detail in Robbins (1978) and Appleby and Oldfield (1992). In brief, ^210^Pb occurs naturally in lake sediments as a radioactive isotope of the ^238^U decay series. In recently-deposited sediments (< 150 years), some of the ^210^Pb activity occurs as a decay product of ^238^U series parents naturally present in the sediments (termed “supported” ^210^Pb). The remaining ^210^Pb activity has originated in the atmosphere by decay of gaseous ^222^Rn and subsequently deposited as “unsupported” fallout ^210^Pb. It is the unsupported, or “excess” fallout ^210^Pb, equivalent to the sediment ^210^Pb activity in excess of the activity of its parent ^226^Ra, that is used to establish a chronology. Once isolated from the atmosphere by burial, excess ^210^Pb is assumed to remain immobile in the sediment column and decay back to supported levels in accordance with its half life (~ 22 years). Under favourable conditions ^210^Pb dating can be used to establish a sedimentation history over a time frame extending up to about 130 years.

Anthropogenic ^137^Cs occurs as a result of fallout from atmospheric nuclear tests undertaken from the mid 1950s to the early 1970s. In the southern hemisphere, the horizon of first appearance of ^137^Cs in the sediment profile dates that horizon between 1954 and 1956, the exact time being a function of the sediment mean particle size, measurement sensitivity, and the core-section thickness (Leslie and Hancock 2008). Due to the low levels of fallout in the southern hemisphere, and the finite time taken to transport sediment-bound ^137^Cs from the catchment to its deposition site, the period of peak fallout (1965) is seldom seen in the sediment record south of the equator (Chaboche et al. 2022). Nevertheless, we prefer to accept the dates ranging between 1954 and 1956 that had been obtained by Leslie and Hancock (2008) as well as Hancock et al. (2011) since our work and those cited were carried out in Australia and on Australian sediments.

The use of ^137^Cs as a chronometer is, therefore, mainly limited to the identification of the mid-1950s time horizon. This horizon is then used to check and calibrate ^210^Pb geochronology.

#### ^210^Pb dating analytical methods

Activities of ^210^Pb and ^137^Cs were determined by gamma spectrometry at the CSIRO Land and Water Laboratories, Canberra (Murray et al. 1987; Leslie 2009) using a well detector and the minimum counting time was 24 h. The oven-dried sediment was homogenised by grinding in a ring mill, mixed with polyester resin and cast into a calibrated geometry. For samples with low mass radiochemical separation procedures and alpha particle spectrometry (Martin and Hancock 2004) were used to determine ^210^Pb (via its ^210^Po daughter) and ^226^Ra. The chemical yield was determined using ^209^Po and ^225^Ra yield tracers.

In addition, the non-monotonic decrease in ^210^Pb activity, and the relatively small catchment area of the lake, the constant rate of supply (CRS) dating model is considered the most appropriate (Appleby and Oldfield 1984).

#### The chronomarker of Pinus pollen in the core sequence

European and American pine trees were introduced to Australia about 150 years ago. *Pinus* pollen grains, belonging mostly to *P. radiata*, became common in the regional pollen rain spectra in Australia after about AD 1880 (Ogden 1996). Pine pollen appears consistently in the top of pollen diagrams throughout southeastern Australia, and Hope (1974) measured pine pollen deposition at 120 grains cm^−2^ year^−1^ at Wilsons Promontory in Victoria. Radiocarbon dating does not provide a reliable indication of the age of first appearance, but the fact that it appears soon after the first signs of European disturbance means that an age of ca AD 1890, or about 30 years after first settlement, is widely accepted. Hope et al. (2019) also recovered *Pinus* pollen in core RNX-A from Rennix Gap Bog site (centred on 36°22.0′S 148°30.2′E) which is located in a sub-alpine peatland in Kosciuszko National Park at an altitude of 1575 m a.s.l.

An age of AD 1890 ± 20 seems likely for Blue Lake, as it collects regional pollen preferentially through snow melt (Raine 1974).

#### Pollen sample preparation

The frozen finger samples were selected at 4 cm intervals with 2 ml of sediment taken for processing. Standard processing utilised hydrofluoric acid digestion of quartz and silicates followed by oxidation with acetolysis to remove organic materials such as humates and cellulose. The pollen was counted from slides with particular attention being paid to finding *Pinus* pollen grains, which are easily distinguished from the local gymnosperm, *Podocarpus*. Pollen percentage diagrams were constructed using a pollen sum of dryland plants (excluding ferns).

#### AMS radiocarbon sample preparation

In the absence of micro-remains such as seeds and grass cuticles, we had to rely on bulk samples for radiocarbon dating. Such an absence is not surprising since the lake is oligotrophic and very dilute in composition (Williams et al. 1970). Bulk samples were physically and chemically pre-treated before being converted to CO_2_. Physical treatment involved the following processes: sieving to eliminate large fractions such as seeds, leaves and pieces of wood; milling to homogenise the fine grained fraction, and the chemical treatment included standard acid–alkali–acid as specified in Hua et al. (2001) and which was done at the ANTARES AMS Centre of the Australian Nuclear and Science and Technology Organisation in Menai, NSW (= New South Wales). The pre-treated samples were then converted to CO_2_ by combustion using the sealed-tube technique of Vandeputte et al. (1996) which is further detailed in Hua et al. (2001). For details on the graphitisation targets technique, refer to Jacobsen et al. (1997). The age calibration was carried out using the OxCal program v.4.4.2 and the SHCal20 data. The calibrated dates reported here σ as median age with 2 standard deviations.

#### Optical dating of sediments

Optical dating can be used to estimate the time elapsed since buried sediment grains were last exposed to sunlight (Huntley et al. 1985; Aitken 1998). This method of sediment dating makes use of the fact that daylight releases charge from light-sensitive electron traps in the defects in crystals such as quartz and feldspar. The release of trapped charge by light resets the optically stimulated luminescence (OSL) signal; this process is commonly referred to as bleaching. When grains of quartz are buried and hidden from light, they begin to accumulate a trapped-charge population due to the effects of ionising radiation, such as that arising from radionuclides naturally present in the deposit. This trapped-charge population increases with burial time in a measurable and predictable way. As a result, the time elapsed since sediment grains were buried can be determined by measuring the OSL signal (burial-dose) from a sample of sediment and estimating the ionising radiation to which it has been exposed since burial (the dose rate) such that

1 $$ {\text{Burial}} \,{\text{time }}\left( {{\text{years}}} \right) \, = \frac{{\text{ Burial}} - {\text{dose }}\left( {{\text{Gy}}} \right)}{{\text{Dose Rate }}\left( {{\text{Gy year}}^{{ - {1}}} } \right)} $$

(Gy = gray).

Optical dating has been successfully used to date aeolian, fluvial and coastal dune sediments, e.g. Bailey et al. (2001), Murray and Clemmensen (2001), Radtke et al. (2001), Hilgers et al. (2001), Olley et al. (1999), and Murray and Olley (2002), as well as deep-sea sediments (Olley et al. 2004).

#### OSL sample preparation

The core consisted of fine-grained organic-rich sediment (loss on ignition 17–28%) with a small amount of sand-sized particles dispersed through the matrix. Sand particles reaching the lake are considered to be aeolian in origin and this is well documented in the study of Stanley and De Deckker (2002). Two sediment samples, from depths of 60–62 cm (BL-1) and 116–118 cm (BL-2), were taken from the core in the laboratory under subdued red illumination. (One AMS date was also obtained for a gyttia from a sediment depth of 114–116 cm, just above the basal OSL sample). Sand grains (90–125 µm or 180–212 µm in diameter) were extracted by wet sieving. In each case, only a small number of sand grains were recovered (BL-1: ~ 30 grains, and BL-2: ~ 100 grains). The quartz grains were then etched in 40% hydrofluoric acid for 50 min to remove the outer 10 µm rinds (Aitken 1985), and to completely remove any feldspars. Acid-soluble fluorides were removed in 15% hydrochloric acid. Samples adjacent to OSL samples were taken for water content determination and for measurement of the lithogenic radionuclide concentrations.

#### OSL analytical methods

The burial dose was determined by OSL measurements from single grains of quartz. All measurements were made on a Risø automated TL/OSL reader, fitted with an EMI 9635QA photomultiplier tube and three U-340 transmission filters. The reader is also equipped with a green-plus-blue (420–550 nm) light source, giving an illumination intensity of about 25 mW cm^−2^ on the sample. The machine has a ^90^Sr/^90^Y beta source, delivering 0.0288 Gy s^−1^ to quartz mounted on stainless steel discs using silicone oil. The experiments were run using Risø TL-OSL software version 4.65.

The single grains were analysed using the regenerative-dose protocol described by Roberts et al. (1998), which was modified from those presented by Murray and Roberts (1998) and by Murray and Mejdahl (1999). Typically, using this protocol, the dose (De) for each grain is calculated as:

2 $$ D_{e} = \, \left( {L_{n} /L_{r} } \right) \, \times \, \left( {T_{2} /T_{1} } \right) \, \times {\text{ regenerative dose}} $$

where L_n_, L_r_, T_1_ and T_2_ are the OSL signals produced by the natural, regenerative, test 1, and test 2 doses, respectively. The test dose signals are used to correct for any changes in OSL sensitivity between the natural (T_1_) and regenerative (T_2_) dose cycles. A test dose ratio of 1 indicates that the analytical protocol has not induced any change in the sensitivity of the grains. The samples were illuminated for 125 s at 125 °C. In each case, the OSL signal was integrated over the first 20 s of illumination, and the OSL signal integrated over the final 20 s was subtracted as background. The reported D_e_ uncertainties are based on the counting statistics, and incorporate calibration uncertainties for the beta sources. A preheat temperature of 240 °C for 10 s was used for L_n_ and L_r_ measurements, and a cut-heat to 160 °C was given after each test dose. In this study, a test dose of 0.6 Gy and regenerative doses of either 3 or 8 Gy were given.

The dose rates were determined from the radionuclide concentrations in the sediment samples collected adjacent to the OSL samples. These samples were analysed by a combination of high-resolution alpha and gamma spectrometry (Murray et al. 1987; Martin and Hancock 2004). Independent checks on calibration were performed using various standards from the US National Bureau of Standards, and IAEA inter-comparisons. The K concentration on the sample from adjacent to BL-2 was determined by X-ray fluorescence analysis. The sample was prepared for analysis by fusing 0.4 g of fine ground sample in a lithium borate glass bead at 1000 °C (Norrish and Hutton 1969; Norrish and Chappell 1977).

Note that all the analytical procedures were performed some 15 years ago using the standard procedures at the time.

## Results

### Stratigraphic correlation of the frozen finger and tonker cores

**Fig. 2:**
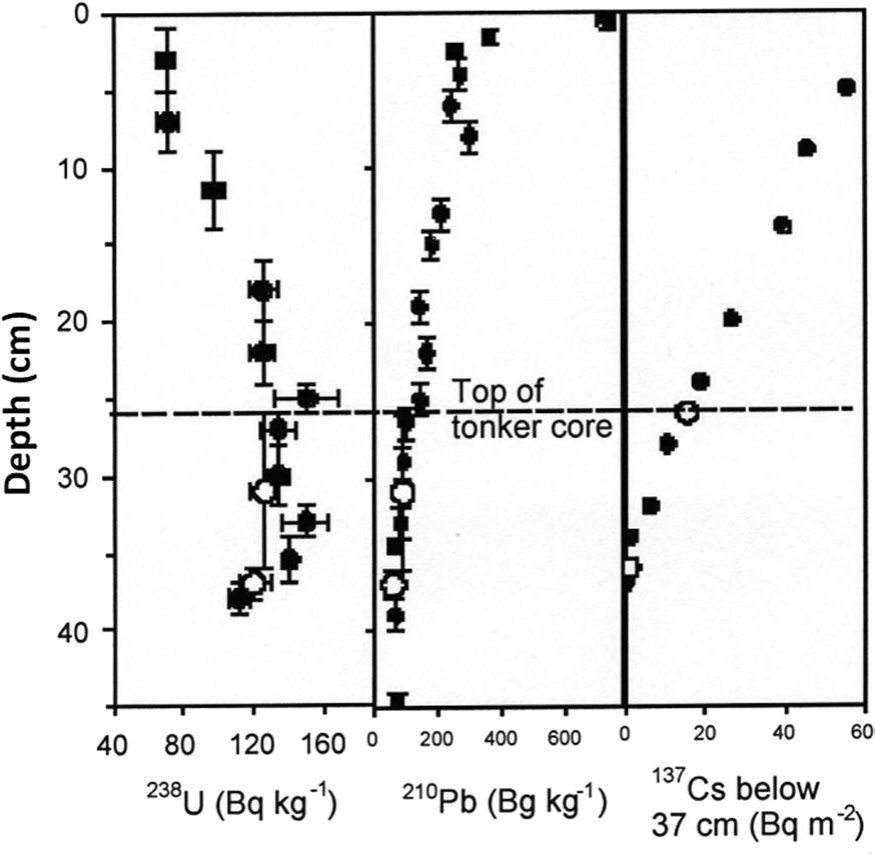
Profiles of ^238^U, ^210^Pb and ^137^Cs inventory above 44 cm. Closed circles represent samples from the frozen finger core, empty circles from the tonker core. The measurements are consistent with the loss of the upper 26 cm of sediment from the tonker core

Radionuclide measurements were used to vertically align the frozen finger and tonker cores. No other stratigraphic information, such as a coloured layer, could be found. Measurements of ^238^U, excess ^210^Pb and ^137^Cs show the most variability in the upper 30 cm (Table 1). The frozen finger profiles are shown in Fig. 2, along with two measurements from the uppermost 13 cm of the tonker core; ^137^Cs is expressed as the inventory of activity above 37 cm (units of Bq m^−2^), 37 cm being the maximum depth of penetration of ^137^Cs activity. All profiles indicate an offset of about 26 cm between the cores, with the tonker core being stratigraphically deeper. This is consistent with the top 26 cm of surface sediment from the tonker core having been lost, a loss that could have easily occurred given the coring mechanism (drop-hammer) and the high porosity of the upper layers of sediment. The frozen finger core on the other hand retained the integrity of porous surficial sediments, with the sediment–water interface being clearly visible.

Based on this evidence the nominal depth of tonker core sections have been increased by 26 cm to create stratigraphic equivalence with the frozen finger core.

### ^210^Pb-^137^Cs chronology

**Fig. 3:**
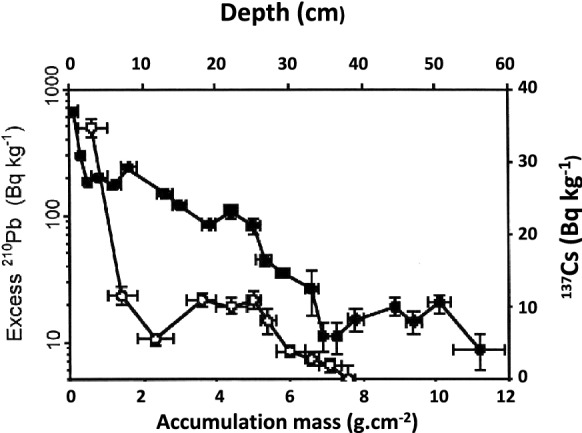
Plots of excess ^210^Pb [closed circles] and ^137^Cs [open circles] activity against depth (accumulated sediment mass) in sediment samples from the frozen finger core collected from Blue Lake in the Snowy Mountains, NSW. Note the log scale for excess ^210^Pb

**Fig. 4:**
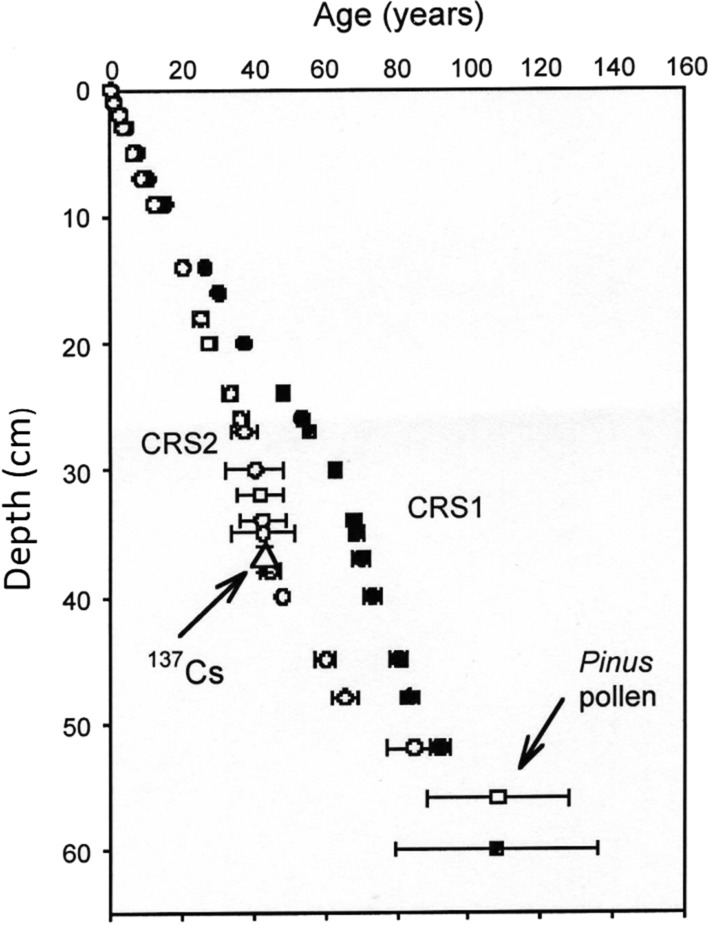
Age versus depth plots for CRS1 (closed circles) and CRS 2 (open circles) as determined from the ^210^Pb_ex_ profile of the frozen finger sediment. Also shown are the chronomarkers used to calculate the CRS2 chronology; ^137^Cs (closed triangle) and *Pinus* (open square)

Plots of excess ^210^Pb (log-linear) and ^137^Cs against depth are shown in Fig. 3. Depth is expressed as cumulative mass (g cm^−2^ yr^−1^) to eliminate the effect of variable water content. Given the non-linearity of the plot in Fig. 4, the non-monotonic decrease in ^210^Pb activity, and the relatively small catchment area of the lake, the constant rate of supply (CRS) dating model is considered the most appropriate (Appleby and Oldfield 1984). With this model, the age of sediment at depth *x* is given by

3 $$ t_{x} = \frac{1}{\lambda }\ln \frac{{A_{0} }}{{A_{x} }} $$

where $A_{0}$ is the total ^210^Pb_ex_ sediment inventory (9130 Bq m^−2^, determined using the tonker core to complete frozen finger ^210^Pb_ex_ profile), and $A_{x}$ is the excess ^210^Pb inventory below depth *x*.

A plot of CRS age and depth (Fig. 4) shows that the calculated age using the conventional CRS approach (labelled CRS1, Eq. 3) agrees with the proxy age provided by *Pinus* pollen (1890 ± 20), but not with the first appearance of ^137^Cs (1955 ± 1 years, as determined from the protocols of Leslie and Hancock (2008). While the potential exists for the mobility of ^137^Cs in organic-rich sediments via desorption from the sediment particles and downward diffusion in porewater (Torgensen and Longmore 1984), we have confirmed the ^137^Cs chronology in this work using measurements of plutonium, a chronomarker with the same origins of ^137^Cs (bomb fallout) but with much greater particle binding characteristics (Sholkovitz and Mann 1984). The total ^137^Cs inventory is 83.9 mBq cm^−2^.

To address the discrepancy between ^210^Pb and ^137^Cs chronologies and improve the chronology of the last few decades a piece-wise CRS approach (labelled CRS2) has been used (Appleby 1992). This approach fixes two or more horizons in the profile with proxy ages determined by other dating methods, and assumes that the ^210^Pb_ex_ flux (*F*) between each pair has been constant, i.e.

4 $$ F = \frac{\lambda \Delta A}{{e^{{ - \lambda t_{1} }} - e^{{ - \lambda t_{2} }} }} $$

where *t*_1_ and *t*_2_ are the ages of the dated horizons at depths *x*_1_ and *x*_2_, and is the ^210^Pb_ex_ inventory between *x*_1_ and *x*_2_. The age of sediment (*t*) at depth *x*, where *x* lies between *x*_1_ and *x*_2_, is given by

5 $$ t = \frac{1}{\lambda }\ln \left( {e^{{ - \lambda t_{1} }} + \frac{\lambda }{F}A_{x} } \right) $$

In applying Eqs. (4) and (5), we have used the dates associated with the first appearance of *Pinus* pollen (1890 ± 10 yrs at 56 cm) and ^137^Cs (1955 ± 1 yr at 35 cm), together with the core surface (1998). Thus, the model assumes two periods of constant (but different) flux, from 1998 to 1955, and from 1955 to 1890. CRS2 ages are compared with CRS1 ages in Fig. 4. Uncertainties are calculated using equations given in Appleby (2001) in combination with the uncertainties associated with the ^137^Cs and *Pinus* chronomarkers (Table 2).

**Table 2:** List of selected horizons and calculated ages with respective justification

Depth* (cm)	Date range	Method
0	0	Assumed from sediment–water interface in frozen finger core
35	1954–1956	^137^Cs, first appearance in core profile
52	1908–1916	^210^Pb CRS2
56	1880–1900	*Pinus* pollen, first appearance in core profile
60–66		951 ± 152 calibrated AMS yrs BP
86–88	~ 1793–1833	OSL
140–142		1211 ± 130 calibrated AMS yrs BP
142–144	~ 1500–1600	OSL

*Depth is corrected for loss of surface material from the tonker core

The dry sediment-accumulation rate (SAR) at time *t* in the past can be calculated from

6 $$ {\text{SAR}} = \frac{{{\text{Fe}}^{ - \lambda t} }}{{C_{x} }} $$

where *C*_x_ is the activity of ^210^Pb_ex_ at depth *x.*

### Pollen results

**Fig. 5:**
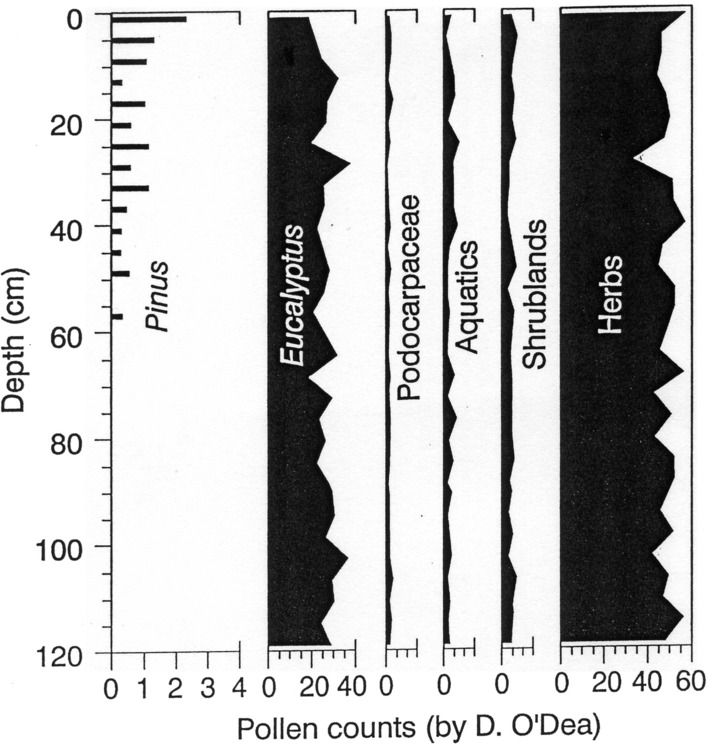
Selected pollen curves for the frozen finger core taken in 1998. *Pinus* is exaggerated × 4 on a dryland pollen sum. Counts performed by D. O’Dea

A summary pollen diagram from the frozen finger core is shown in Fig. 5. The pine-pollen curve is shown at four times the measured values. The deepest occurrence of *Pinus* occurs in the frozen finger core at 56 cm depth, although none was found at 52 cm, above this there is continuous and increasing presence from 48 cm to the surface. We have assigned an age of 1890 ± 20 AD to the 56 cm horizon.

### OSL dates

#### Dose rates

**Fig. 6:**
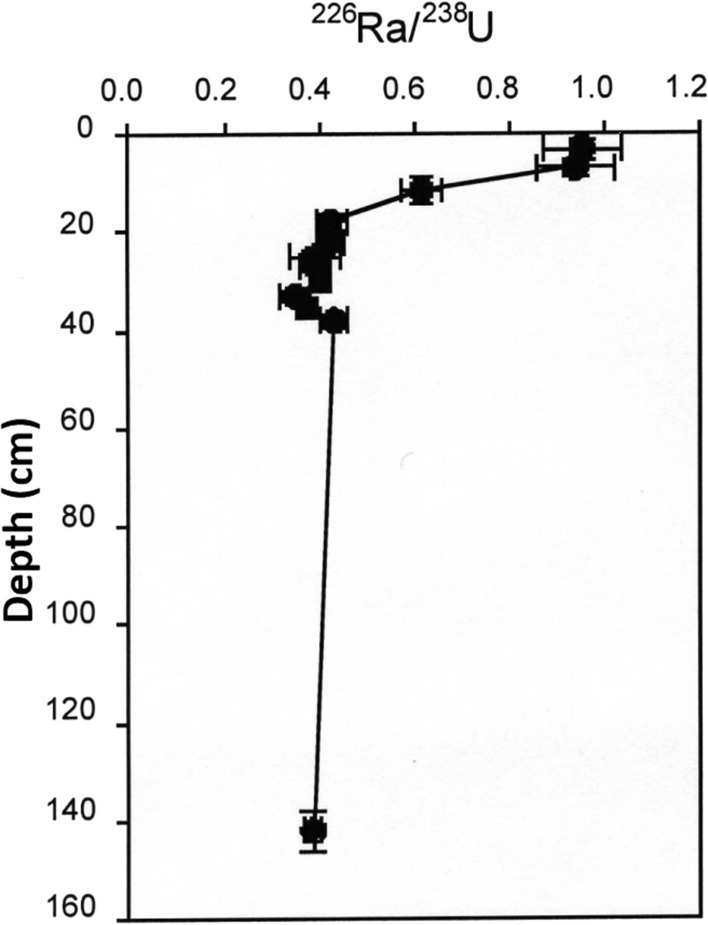
The ^226^Ra/^238^U ratio against depth in sediment samples from the tonker core collected from Blue Lake. Horizontal error bars indicate ± 1 standard error; vertical bars represent the depth interval

The frozen finger lithogenic radionuclide data (Table 1) and the tonker data (Table 3) show a measured disequilibrium in the upper part of the uranium decay series. Figure 6 shows that the ^226^Ra/^238^U ratio close to one in the upper 7 cm, but decreases to an average value of around 0.40 at 18 cm, and remains close to that value down to 146 cm. In the sample from 138 to 146 cm ^226^Ra and ^230^Th activity concentrations are consistent with secular equilibrium. We assume this to be the case for all sediment and attribute the disequilibrium between ^226^Ra and ^238^U to uranium addition following deposition. Using the ^210^Pb dates and the data presented in Fig. 2, we infer that this addition occurs at constant rate for the first 25 years following burial, at which time the ^226^Ra/^238^U activity ratio reaches a value of 0.4. This has been used to produce time-dependent corrections to the dose rate. Dose rates were calculated using the conversion factors of Olley et al. (1996) and the computer program listed in Roberts et al. (1993). The water content measured in the samples collected adjacent to the OSL samples were consistent, and an average value of 78 ± 5% of the dry weight. This has been used to correct the dry dose rate for water content following Aitken (1985). The time-dependent changes in the down core water content are small, however we have incorporated the effects into the dose-rate calculations.

**Table 3:** Tonker core activity concentrations (Bq kg^−1^) of lithogenic radionuclides used for OSL dose rate calculation and stratigraphic correlation with frozen finger core. Uncertainties correspond to 1 standard deviation

Observed depth (cm)	Corrected depth (cm)	^137^Cs	^238^U	^234^U	^230^Th	^226^Ra	^210^Pb	^232^Th	^40^K
0–10	26–36	7.6 + 0.7	127 + 8			44.6 + 1.6	88.3 + 8.0	57.2 + 2.3	582 + 16
10–30	36–38	0.2 + 0.3	138 + 17					58.7 + 2.7	550 + 20
59–60	85–86					54.9 + 2.3	50.3 + 1.5		
112–120	138–146		125 + 4	158 + 5	47.0 + 1.7	51 + 4	60 + 2	55 + 1.9	560 + 10

The cosmic ray dose rates were calculated from Prescott and Stephan (1982) and Prescott and Hutton (1988). Beta-attenuation factors were taken from Mejdahl (1979) and the effective alpha dose rate contribution has been estimated using an alpha-efficiency ‘a’ value for quartz of 0.04 ± 0.02. The alpha dose rate contribution is about 7% of the total dose rate. The calculated total dose rates are 2.33 ± 0.17 and 2.34 ± 0.17 mGy y^−1^ for samples BL-1 and BL-2, respectively.

#### Dose in single grains

**Fig. 7:**
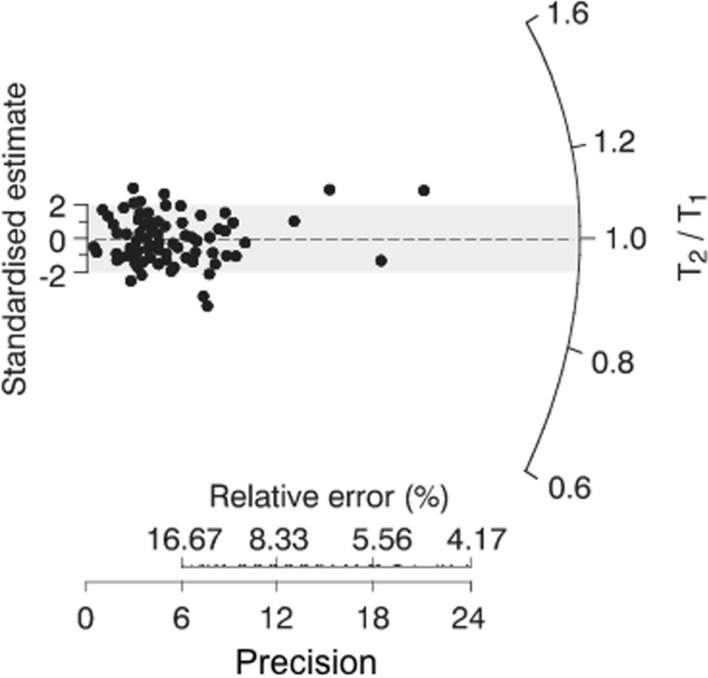
Radial plot of the test dose ratios for single grains of quartz from samples BL-1 and BL-2, from Blue Lake

The test-dose ratios for the individual grains of quartz from both samples are presented in a radial plot in Fig. 7. The ratio measured for a grain can be read by drawing a line from the y-axis origin through the point until the line intersects the radial axis (log scale) on the right-hand side. Its standard error can be read by extending a line vertically to intersect the x-axis. The x-axis has two scales: one plots the relative standard error of the ratio (in %) and the other (precision) plots the reciprocal standard error of the log estimate. Therefore, values with the highest precisions and the smallest relative errors plot closest to the radial axis on the right of the diagram, and the least precise estimates plot furthest to the left.

The y-axis provides a further aid to data display, by plotting standardised estimates of the log ratio. These are calculated by subtracting a reference value (such as the pooled log ratio for all aliquots, or another log ratio of interest; in this case a value of 1 was used) from each of the log ratios and then dividing each of the differences by the associated standard error. A useful feature of the standardised estimate is that ratios that are statistically concordant at the 2 confidence level will fall within a band extending ± 2 units vertically about a common radial line. That is, if the individual ratio estimates from a sample are consistent with a common ratio, then 95% of the points should lie within a ± 2 band. Galbraith et al. (1999) provide further details, and a worked illustration, of how radial plots may be used to display OSL data.

The test-dose ratio for most of the grains is consistent with a value 1 (central value 0.98 ± 0.03). Consequently, with the exception of the four individual grains which have test-dose ratios significant different to 1 and standard errors of < 20%, we have assumed a common test-dose ratio of 1. This assumption means that more of the data can be included in the following analysis, but it does not significantly affect the burial-age calculations.

**Fig. 8:**
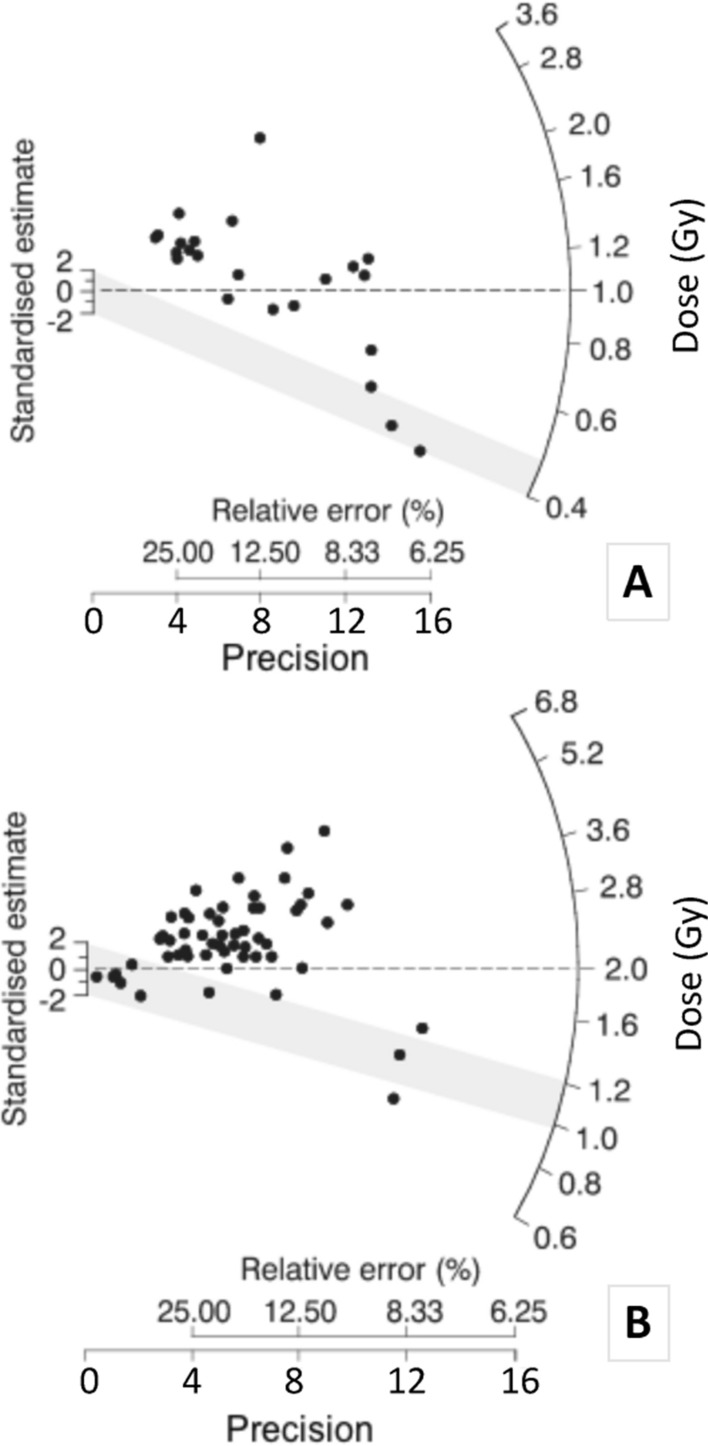
Radial plot of measured doses in A for 24 single grains from sample BL-1. The shaded region shows the calculated burial dose; in B for radial plot of measured doses for 55 single grains from sample BL-2. The shaded region shows the calculated burial dose

The doses measured in the individual grains from sample BL-2 are presented in a radial plot in Fig. 8b. The measured doses range from 0.83 ± 0.07 to 9 ± 2 Gy. This spread in dose suggests that not all of the grains were fully bleached at the time of burial. In such circumstances, the best estimate of the burial dose will be provided by the grains containing the lowest doses (Olley et al. 1999). Consequently, the burial dose for this sample has been calculated using the lowest dose population; this consists of 10 grains, which include the three grains with the highest precision-dose estimates (Db = 1.10 ± 0.06 Gy). This group of samples may still yield an over estimate of the burial age, because the grains with the lowest dose may also not have been fully bleached at burial.

Doses measured in single grains from sample BL-1 ranged from 0.39 ± 0.03 to 5.8 ± 0.7 Gy (Fig. 8a). Of the 30 grains recovered from the core sample, only 24 grains gave measurable OSL signals. The burial dose has been estimated from the three lowest measured doses; these estimates are those calculated with the highest precision (better than 10%), but we cannot be certain that even these grains were fully bleached at deposition (Db = 0.43 ± 0.03 Gy).

#### Burial ages

Using Eq. (2), we estimate the maximum burial age for sample BL-1 (corrected tonker depth 86–88 cm) is 185 ± 20 years, and for BL-2 (corrected depth 142–142 cm) the maximum burial age is 470 ± 50 years. As noted earlier, we need to add 26 cm to those depths if we are to provide ages for sediments below the lake floor, due to the apparent loss of sediment from the tonker core.

### AMS radiocarbon dates

The sediment horizons from which the samples were taken for radiocarbon dating have conventional radiocarbon ages of 1090 ± 60 years BP (OZD 730) and 1350 ± 60 (OZD 731) respectively. These give calibrated mean ages with best estimates of 1861 ± 154 (2 σ) years BP for OZD 730 and 1211 ± 130 (2 σ) years BP for OZD 731. However, these samples were corrected for tonker depths of 61–62 cm and 140–142 cm, respectively.

## Discussion

**Fig. 9:**
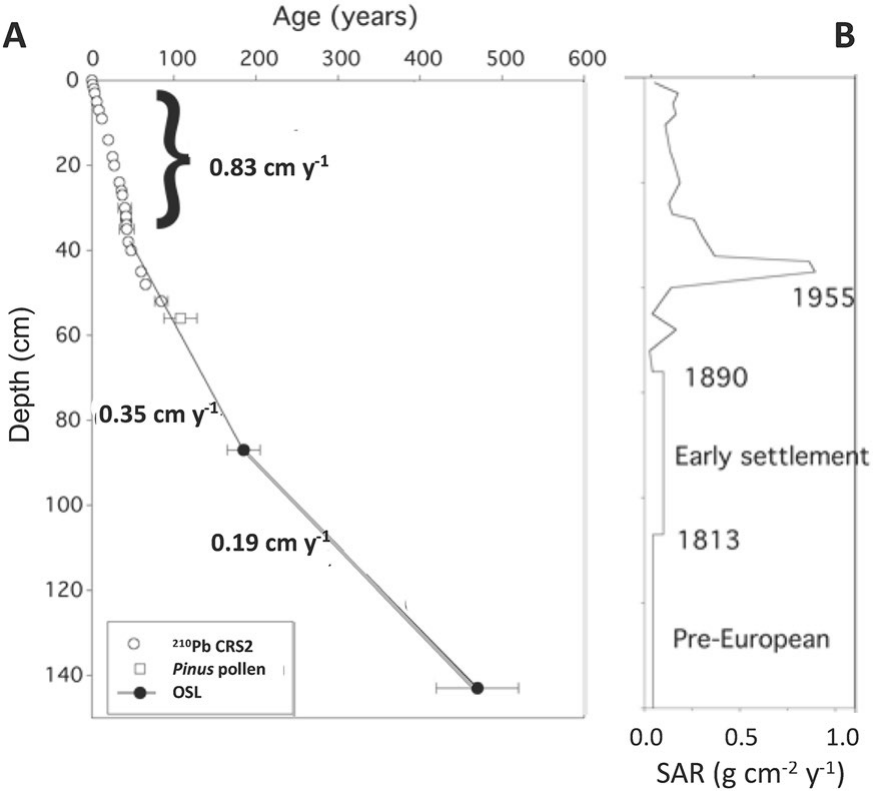
Summary diagram for sediment core samples collected from Blue Lake in the Snowy Mountains.** A** Age versus depth model. **B** Sediment accumulation rate,* r*_*t*_ (determined using the CRS model), versus depth

The deposition ages inferred from the ^210^Pb and OSL dating, and the first appearance of *Pinus* pollen are shown against core depth in Fig. 9. As indicated above, tonker core depths have had 26 cm added due to loss of the upper portion of the core. Also shown in Fig. 9 is the sedimentation accumulation rate in cm yr^−1^ and the dry sediment mass accumulation rate in g cm^−2^ yr^−1^ as a function of depth. The chronology summary can be subdivided into three main periods of sedimentation; a pre-European phase (pre- ~ 1813), an early European settlement phase (~ 1813–1900) and an expanded European settlement phase from ~ 1900 onwards.

The pre-European SAR is estimated from the two OSL dates at the corrected tonker depths of 86–88 cm (185 ± 20 yrs) and 142–144 cm (470 ± 50 yrs). A SAR of 0.19 ± 0.04 cm yr^−1^ (equivalent to 0.046 ± 0.010 g cm^−2^ yr^−1^) is calculated for the ~ 300 year period prior to 1813. A higher SAR (0.35 ± 0.08 cm yr^−1^) is calculated for the early settlement period, as constrained by the most recent OSL date (1813 ± 20 yrs) at 86–88 cm and the oldest CRS2 ^210^Pb date (1912 ± 3 yrs) at 52 cm. For the post-1900 period ^210^Pb-derived CRS2 dates indicate a mean SAR of 0.60 ± 0.03 cm yr^−1^, a rate three times higher than the pre-European rate. Figure 9 (inset) shows that within the 1900s great variation is seen with the SAR rising rapidly from ~ 1940 onwards and peaking in the early-mid 1950s at a value 18 times the pre-European rate. SARs then decrease from the 1960s onwards back to rates seen in the early 1900s.

The New South Wales Government Botanist in 1898 described with some alarm the extensive erosion that occurred in the highlands when drought forced stockmen to remain in the area with sheep and cattle (Maiden 1899). This was followed by Byles' report (1932; reference in Costin et al. 1979) describing significant erosion and vegetation destruction in the Kosciuszko region. While this history correlates well with the approximately two times increase in SARs calculated from OSL and pollen dates for the 1800s and early 1900s, it appears that other factors must have accelerated erosion during the1940-1960 period. A combination of a period of low rainfall in southeastern Australia in the 1930s and 1940s followed a relatively wet period in the 1950s (http://www.bom.gov.au/jsp/ncc/cdio/weatherData/av?p_nccObsCode=139&p_display_type=dataFile&p_stn_num=071021) (Glochinbah at 990 m a.s.l.) with 1153.6 mm in 1950 and 1054.0 mm in 1952 compared to the mean for 1907–2021 being 631.1 mm could explain this observed increase of SAR. Soils exposed by vegetation destruction by overgrazing would have been susceptible to erosion by heavy rainfall in summer (in 1950: 452.0 mm in February and March alone combined as recorded at Glochinbah). Thus, the increase in the SAR in the upper part of the Blue Lake core is attributed to the onset of sheep and cattle grazing. Grazing by livestock ceased in the catchment in 1967. There is evidence that this land-use change may have had an effect on accumulation rates since the late 1980s, with a decrease towards pre-European rates being seen in the upper 2 cm (Fig. 9B). However, a sustained decrease would need to be seen before this trend could be confirmed.

The inferred deposition ages from the ^210^Pb and OSL dating, and the first appearance of pine pollen (Fig. 8), are significantly younger than the radiocarbon-age estimates. This suggests that the organic remains used in the radiocarbon dating may have been ‘stored’ in the catchment for a significant period prior to their final transport and subsequent deposition in the lake. Walker et al. (2000) estimated a storage time of up to 400 years for organic matter using measurements of ^14^C in sediment from a tropical crater lake. The organic matter was derived mainly from decomposing trees, and the storage time included the age of the tree (about 200 years) and the decomposition time in the marginal shallows (estimated at 100–200 years). In alpine environments, where plant decomposition rates may be much slower, greater storage times are possible. Costin (1972), in his overview of the ^14^C ages for numerous deposits in the Snowy Mountains area, clearly identified some solifluction deposits in the Mount Twynam northeastern cirque located just above and upstream of Blue Lake. He also recognised exposed deposits “along the side of a creek that now drains what was formally a glacial lake” and obtained a radiocarbon date of 2520 ± 160 years BP for the base of a snow patch peat. He also dated another peat on bedrock on the headwall of that cirque as 2290 ± 110 years BP (both being conventional radiocarbon dates). Thus, erosion caused in the catchment could have supplied a substantial amount of old carbon into Blue Lake. Costin (1972) also lists much older deposits that are visible in the area that would have supplied carbon to the lowest parts of the landscape (viz. Blue Lake) around 2500 years BP when peats formed during a cold phase. Solifluction terraces are common in the area and Costin (1972) assembled ages for those which range between 2250 and 2980 years BP.

Our paper clearly illustrates that there is a call for caution when dating lake sediments from alpine environments. Radiocarbon is a technique that is frequently applied to dating lacustrine, organic-rich material, but one needs to pay attention to the fact that older material can readily enter a lake and, therefore, “contaminate” the organic material engendered in the lake. This also applies to organic compounds such as humic acids generated from outside the lake, which can be generated for older carbonaceous material. This is particularly the case in alpine environments where vegetational growth is slow, and where the age of some of the vegetation can span many centuries.

The data on aeolian quartz grains recovered from a Blue Lake core by Stanley and De Deckker (2002), which has implications for the timing of an increase in stronger winds [and considered to indicate aridity] in the second part of the Holocene, needs to be re-examined with respect to its chronology. Nevertheless, the onstart of aridity reconstructed by Gingele et al. (2007) from a core offshore the mouth of the River Murray in South Australia coincides well with the record of Blue Lake. In addition, De Deckker (2022) in his recent review of the Holocene of eastern Australia, identified that the timing of the warm phase recognised in the core from Blue Lake by Raine (1974)—with the occurrence of the pollen of *Pomaderris aspera*—coincides well with other warm and wet events at many parts of eastern Australia. It may be that at the end of the wet Holocene period, the vegetation cover in the catchment of Blue Lake was sufficient enough to prevent ‘old’ carbon to trickle into the lake itself. This needs to be verified.

The recent catastrophic fires of January 2003 that burned a substantial amount of heath and other vegetation in the catchment of Blue Lake would have surely triggered slope instability and engendered once again an increase in sediment accumulation in the lake, and introduced once more a significant amount of ‘old’ carbon into the lake, including charcoal.

## Conclusions

The results presented here bring into question the veracity of radiocarbon dating particularly in alpine environments, and illustrate the need to use several dating techniques to determine, with confidence, the timing of deposition of lacustrine sediments. These results also identify that sedimentation in Blue Lake has increased by a factor of two after European settlement, and reached a rate seven times higher in the mid 1950s. This is attributed to increased erosion caused by sheep and cattle grazing in the lake’s catchment, in addition to increased rainfall. Our work also indicates that since removal of cattle from the lake’s catchment erosion rates have diminished and have returned to levels more comparable to those that existed prior to European land-use practices in the catchment.

This study demonstrates that OSL dating can be applied successfully to single grains of quartz deposited in a lacustrine environment for less than 500 years.

Finally, compared to the millennial changes recorded in the two Italian and French lakes in the European Alps that are linked to the introduction of livestock, it is clear that erosion did occur almost immediately in the catchment of Blue Lake. By analogy, the same erosive effects must have occurred everywhere in the Australian Alps. Luckily, the erosive and vegetational changes were halted after livestock was withdrawn from the Australian Alps. Let us hope that the current feral equine presence in parts of the National Park that are well known to affect the soils and vegetation [https://invasives.org.au/our-work/feral-animals/feral-horses/] will soon cease as a result of future governmental decisions.

## References

[CR1] Aitken MJ (1985). Thermoluminescence dating.

[CR2] Aitken MJ (1998) An introduction to optical dating. In: The dating of Quaternary sediments by the use of photon-stimulated luminescence, Oxford University Press, Oxford

[CR3] Appleby PG, Oldfield F (1984). The assessment of ^210^Pb data from sites with varying sediment accumulation rates. Hydrobiologia.

[CR4] Appleby PG, Oldfield F, Ivanovich M, Harmon RS (1992). Application of lead-210 to sedimentation studies. Uranium-series disequilibrium: applications to Earth.

[CR5] Bailey SD, Wintle AG, Duller GAT, Bristow CS (2001). Sand deposition during the last millenium at Aberffraw, Anglesey, North Wales as determined by OSL dating of quartz. Quatern Sci Rev.

[CR6] Bajard M (2017). Long-term changes in alpine pedogenetic processes: effect of millennial agro-pastoralism activities (French-Italian Alps). Geoderma.

[CR7] Bajard M (2020). Pastoralism increased vulnerability of a subalpine catchment to flood hazard through changing soil properties. Palaeogeogr, Palaeoclimatol, Palaeoecol.

[CR8] Barrows TT, Stone JO, Fifield LK, Creswell RG (2001). Late Pleistocene glaciation at the Kosciusko Massif, Snowy Mountains, Australia. Quat Res.

[CR9] Berglund BE (1986). Handbook of holocene palaeoecology and palaeohydrology.

[CR10] Bowler JM, Huang Q, Kezao C, Head MJ, Baoyin Y (1986). Radiocarbon dating of playa-lake hydrologic changes: examples from northwestern China and central Australia. Palaeogeogr Palaeclimatol Palaeoecol.

[CR12] Burgess J, Gillieson D, Spate A (1988). On the thermal stratification of freshwater lakes in the Snowy Mountains, Australia, and the Larseman Hills, Antarctica. Search.

[CR13] Byles BU (1932). A reconnaissance of the mountainous part of the River Murray catchment in New South Wales. Forestry Bur Bull.

[CR14] Chaboche P-A (2022). ^240^Pu/^239^Pu signatures allow refining the chronology of radionuclide fallout in South America. Sci Total Environ.

[CR15] Costin AB (1972). Carbon-14 dates from the Snowy Mountains area, southeastern Australia, and their interpretation. Quat Res.

[CR16] Costin AB, Gray M, Todderdell CJ, Wimbush DJ (1979). Kosciusko Alpine Flora.

[CR17] David TWE, Helm R, Pittman EF (1901). Geological notes on Kosciusko, with special reference to evidences of glacial action. Proc Linn Soc NSW.

[CR18] De Deckker P (2022). The Holocene hypsithermal in the Australian region. Quat Sci Adv.

[CR19] De Deckker P, Moros M, Perner K, Blanz T, Wacker L, Schneider R, Barrows TT, O’Loingsigh T, Jansen E (2020). Climatic evolution in the Australian region over the last 94 ka - spanning human occupancy, and unveiling the Last Glacial Maximum. Quat Sci Rev.

[CR21] Dulhunty JA (1945). On glacial lakes in the Kosciusko region. J R Soc NSW.

[CR22] Galbraith RF, Roberts RG, Laslett GM, Yoshida H, Olley JM (1999). Optical dating of single and multiple grains of quartz from Jinmium rock shelter, northern Australia. Part I: experimental design and statistical models. Archaeometry.

[CR23] Galloway RW (1963). Glaciation in the Snowy Mountains: a reappraisal. Proc Linn Soc NSW.

[CR24] Gillespie R, Magee JW, Luly JG, Sparks RJ, Wallace G (1991). AMS radiocarbon dating in the study of arid environments: examples from Lake Eyre, South Australia. Palaeogeogr Palaeclimatol Palaeoecol.

[CR71] Gingele FX, De Deckker P, Norman M (2007) Late Pleistocene and Holocene climate of SE Australia reconstructed from dust and river loads deposited offshore the River Murray Mouth. Earth Planet Sci Lett 255:257–272

[CR25] Good R (editor) (1989) The scientific evidence of the Australian Alps. In Proceedings of the first fenner conference. Australian Academy of Science, Canberra, pp 392

[CR26] Hancock G, Leslie C, Everett S, Tims S, Brunskill G, Haese R (2011). Plutonium as a chronomarker in Australian and New Zealand sediments: a comparison with ^137^Cs. J Environ Radioact.

[CR27] Hilgers A, Murray AS, Schlaak N, Radtke U (2001). Comparison of quartz OSL protocols using Lateglacial and Holocene dune sands from Brandenburg, Germany. Quat Sci Rev.

[CR28] Hope GS (1974). The vegetation history from 6000 B.P. to present of Wilsons Promontory, Victoria. Aust New Phytol.

[CR29] Hope G, Mooney S, Allen K, Baker P, Keaney B, Kemp J, Martin L, Pearson S, Stevenson J, Zheng X (2019). Science through time: understanding the archive at Rennix Gap Bog, a sub-alpine peatland in Kosciuszko National Park, New South Wales, Australia. Proc Linn Soc NSW.

[CR30] Hua Q, Jacobsen GE, Zoppi U, Lawson EM, Williams AA, Smith AM, McGann MJ (2001) Progress in radiocarbon target preparation at the ANTARES AMS Centre. In Proceedings of the 17th International Radiocarbon Conference, Jerusalem, June 18–23, 2000. Radiocarbon 43: 275–282.

[CR31] Huntley DJ, Godfrey-Smith DI, Thewalt MLW (1985). Optical dating of sediments. Nature.

[CR32] Jacobsen G, Hua Q, Tarshishi J, Fink D, Hotchkis MAC, Lawson EM, Smith AM, Tuniz C (1997) AMS radiocarbon analysis of microsamples. In: Handbook of the Sixth Australasian Archaeometry Conference, 10–13 Feb 1997, Sydney, Australia, p 36

[CR33] Leslie C (2009) Analysing environmental radioactivity in soils and sediments using high-purity germanium gamma detectors at CSIRO land and water: procedures and protocols. CSIRO Land and Water Science Report 12/09 CSIRO, Canberra. Available at http://www.clw.csiro.au/ publications/science/2009/sr12–09.pdf

[CR34] Leslie C, Hancock GJ (2008). Estimating the date corresponding the horizon of the first detection of ^137^Cs and ^239+240^Pu in sediment cores. J Environ Radioact.

[CR36] Maiden JH (1899) A contribution towards a flora of Mount Kosciusko. In Agricultural Gazette of N.S.W, Vol 10, pp 1001–1042

[CR37] Martin P, Hancock GJ (2004) Routine analysis of naturally occurring radionuclides in environmental samples by alpha-particle spectrometry*.* In Supervising Scientist Report 180, Supervising Scientist, Darwin, NT, Australia

[CR38] Mejdahl V (1979). Thermoluminescence dating: beta-dose attenuation in quartz grains. Archaeometry.

[CR39] Muller RA (1977). Radioisotope dating with a cyclotron. Science.

[CR40] Murray A, Clemmensen LB (2001). Luminescence dating of Holocene aeolian sand movement, Thy, Denmark. Quart Sci Rev (quat Geochronol).

[CR41] Murray AS, Mejdahl V (1999). Comparison of regenerative-dose single-aliquot and multiple-aliquot (SARA) protocols using heated quartz from archaeological sites. Quat Sci Rev.

[CR42] Murray AS, Olley JM (2002). Precision and accuracy in the optically stimulated luminescence dating of sedimentary quartz: a status review. Geochronometria.

[CR43] Murray AS, Roberts RG (1998). Measurement of the equivalent dose in quartz using a regenerative-dose single-aliquot protocol. Radiat Meas.

[CR44] Murray AS, Marten R, Johnston A, Martin P (1987). Analysis for naturally occurring radionuclides at environmental concentrations by gamma spectrometry. J Radioanal Nucl Chem.

[CR46] Neale JW, Walker D (1996). Sampling sediment under warm deep water. Quat Sci Rev.

[CR47] Norrish K, Chappell BW, Zussman J (1977). X-ray fluorescence spectrometry. Physical methods in determinative mineralogy.

[CR48] Norrish K, Hutton JT (1969). An accurate X-ray spectrographic method for the analysis of a wide range of geological samples. Geochim Cosmochim Acta.

[CR49] Ogden RW (1996) The effects of farming and river regulation on billabongs of the eastern Murray River and lower Ovens River. Unpublished PhD thesis, The Australian National University, Canberra

[CR50] Olley JM, Murray AS, Roberts RG (1996). The effects of disequilibria in the uranium and thorium decay chains on burial dose rates in fluvial sediments. Quat Sci Rev.

[CR52] Olley JM, Caitcheon GG, Roberts RG (1999). The origin of dose distributions in fluvial sediments, and the prospect of dating single grains of quartz from fluvial deposits using Optically stimulated luminescence. Radiat Meas.

[CR53] Olley JM, De Deckker P, Roberts RG, Fifield LK, Yoshida CH, Hancock G (2004). Optical dating of deep-sea sediments using single grains of quartz: a comparison with radiocarbon. Sed Geol.

[CR54] Prescott JR, Hutton JT (1988). Cosmic ray and gamma ray dosimetry for TL and ESR. Nucl Tracks Radiat Meas.

[CR55] Prescott JR, Stephan LG (1982). The contribution of cosmic radiation to the environmental dose for thermoluminescence dating: latitude, altitude and depth dependencies. PACT.

[CR56] Radtke U, Janotta A, Hilgers A, Murray AS (2001). The potential for OSL dating Lateglacial and Holocene dune sands with independent age control of the Laacher See tephra (12880 a) at the Section ‘Mainz-Gonsenheim’. Quat Sci Rev.

[CR57] Raine JI (1974). Pollen sedimentation in relation to Quaternary vegetation history of the Snowy Mountains of New South Wales. Unpublished PhD thesis, The Australian National University, Canberra.

[CR58] Raine JI (1982). Dimictic thermal regime and morphology of Blue Lake in the Snowy Mountains. Aust J Mar Freshw Res.

[CR59] Robbins JA, Nriagu JO (1978). Geochemical and geophysical applications of radioactive lead. The biogeochemistry of lead in the environment Part A.

[CR60] Roberts RG, Uren CJ, Murray AS (1993) Thermoluminescence dating techniques at the Alligator Rivers Region research institute. In Technical memorandum 41, office of the supervising scientist, Australian Government Publishing Service, Canberra

[CR61] Roberts R, Bird M, Olley JM, Galbraith R, Lawson EW, Laslet G, Yoshida H, Jones R, Fullagar R, Jacobsen G, Hua Q (1998). Optical and radiocarbon dating at Jinmium rock shelter in northern Australia. Nature.

[CR62] Scherrer P, Pickering CM (2005). Recovery of Alpine vegetation from grazing and drought: data from long-term photoquadrats in Kosciuszko National Park, Australia. Arct Antarct Alp Res.

[CR63] Sholkovitz ER, Mann DR (1984). The pore water chemistry of ^239,240^Pu and ^137^Cs in sediments of Buzzards Bay, Massachusetts. Geochim Cosmochim Acta.

[CR64] Stanley S, De Deckker P (2002). A Holocene record of allochthonous, aeolian mineral grains in an Australian alpine lake: implications for the history of climate change in southeastern Australia. J Paleolimnol.

[CR65] Timms BV (1979). The benthos of Kosciusko glacial lakes. Proceedings of the Linnean Society of NSW.

[CR66] Torgensen T, Longmore ME (1984). ^137^Cs diffusion in the highly organic sediment of Hidden Lake, Fraser island, Queensland. Aust J Mar Freshw Res.

[CR67] Vandeputte K, Moens L, Dams R (1996). Improved sealed-tube combustion of organic samples to CO_2_ for stable isotopic analysis, radiocarbon dating and percent carbon determinations. Anal Lett.

[CR68] Walker D, Head MJ, Hancock GJ, Murray AS (2000). Establishing a chronology for the last 1000 years of laminated sediment accumulation at Lake Barrine, a tropical upland Maar lake, northeastern Australia. The Holocene.

[CR69] Williams WD, Walker KF, Brand GW (1970). Chemical composition of some inland surface waters and lake deposits of New South Wales. Aust J Mar Freshw Res.

